# Use of Musical Intervention in the Pediatric Intensive Care Unit of a Developing Country: A Pilot Pre–Post Study

**DOI:** 10.3390/children9040455

**Published:** 2022-03-24

**Authors:** Federica Buzzi, Nizar Bakir Yahya, Simone Gambazza, Filippo Binda, Alessandro Galazzi, Antonella Ferrari, Stefano Crespan, Hevan Adel Al-Atroushy, Barbara Maria Cantoni, Dario Laquintana, Collaborative Group

**Affiliations:** 1 Pediatric Unit, Istituto Scientifico, Universitario San Raffaele, 20132 Milan, Italy; buzzi.federica@hsr.it; 2 EU Project MADAD, Italian Association for Solidarity among Peoples, Duhok 42001, Iraq; 3 Hospital Direction, Hevi Pediatric Teaching Hospital, Duhok 42001, Iraq; nezarr11@yahoo.com; 4 Department of Healthcare Professions, Fondazione IRCCS Ca’ Granda Ospedale Maggiore Policlinico, 20122 Milan, Italy; simone.gambazza@policlinico.mi.it (S.G.); filippo.binda@policlinico.mi.it (F.B.); barbara.cantoni@policlinico.mi.it (B.M.C.); dario.laquintana@policlinico.mi.it (D.L.); 5 Curadelsuono432 Project, 30020 Venice, Italy; ella@curadelsuono432.com (A.F.); shantam@curadelsuono432.com (S.C.); 6 Pediatric Intensive Care Unit, Hevi Pediatric Teaching Hospital, Duhok 42001, Iraq; hevanadel6@gmail.com

**Keywords:** musical intervention, nursing care, pain management, comfort behavior scale, developing countries, pediatric intensive care unit

## Abstract

Music is frequently used in different clinical settings, and it is implemented as a complementary, low-cost and useful intervention to reduce pain, anxiety and to improve relaxation. This pilot pre–post study aimed to examine the feasibility and preliminary effectiveness of a specific musical intervention in patients ≤16 years admitted to the Pediatric Intensive Care Unit (PICU) of an Iraqi hospital. The COMFORT Behavior Scale (CBS) was used by nurses to assess the level of sedation. Fifty-nine children were enrolled during the study period (March 2020–August 2021). CBS was lowered by 2.2 (95% CI: 1.9 to 2.6) points after 30 min, and by 3.3 (95% CI: 2.9 to 3.6) points after 60 min from music initiation. Thirty minutes after music initiation, heart rate decreased by 6.3 (95% CI: 4.5 to 8.1) beats per minute, whereas at 60 min, heart rate decreased by 9.1 (95% CI: 7.2 to 10.9) beats per minute. No clinically significant variations were detected in the other vital signs (blood pressure, respiratory rate and oxygen saturation). These findings support the feasibility of musical intervention in a developing country. CBS and heart rate variation may be worth following up in larger and conclusive studies.

## 1. Introduction

Listening to music has been used for centuries by various cultures and professions for a wide range of healing and wellness purposes [1]. Music is used in clinical settings around the world, it is perceived as a complementary, non-invasive, inexpensive and useful intervention to reduce pain, anxiety and distress and to improve relaxation [2,3]. 

In pediatric healthcare, complementary care methods such as the application of musical intervention is a promising resource to be integrated with standard medical treatments for patient recovery and well-being [4,5]. In the Pediatric Intensive Care Unit (PICU), physical environments are constantly saturated with sounds and noises, that can lead to abnormal physiological responses, such as increased heart rate (HR) and/or respiratory rate (RR) [6,7]. Sensory and environmental deprivation, which may characterize ICUs with inadequate staffing or resources, may lead to stressful responses as well, which can be harmful to the neurodevelopment of children during PICU stay [8,9]. Several randomized trials have investigated the impact of musical intervention in critically ill adults and neonates but very few studies have explored musical intervention in pediatric critically ill patients [10]. Furthermore, research is usually conducted in developed countries, often in tertiary level hospitals, whereas literature lacks studies from developing countries, where there are a low number of critical care specialists, access to services can be troublesome, the technological resources are scarce [11] and behavioral responses to musical intervention can be different. 

Therefore, the aim of this study was to examine the implementation of a specific musical intervention in children admitted to PICU in an Iraqi hospital and to search for clinically meaningful variation that may be worth following up in a larger study. 

## 2. Materials and Methods

### 2.1. Study Design and Setting

This pilot study took place at Hevi Pediatric Teaching Hospital of Duhok City (Iraq), from March 2020 to August 2021. All patients aged ≤16 years and admitted to PICU were exposed to a specific musical intervention, twice a day, which was reported using current recommendations [12]. The Hevi Pediatric Teaching Hospital has 190 beds (10 beds in PICU) and it is the main hospital in Duhok, which is the capital city of Duhok Governorate in the Kurdistan Region (Iraq). The Duhok Governorate has received the largest influx of displaced population, both refugees and migrants, since the beginning of the Syrian crisis in 2011 [13]. This phenomenon has put pressure on the health care system and economy given that the hosting capacities are overstretched. 

The study protocol was approved by the Ethics Committee of Hevi Paediatric Teaching Hospital of Duhok.

### 2.2. Musical Intervention

Music was composed, played and produced by two musicians and music therapists. Music used precise technical criteria, which did not consider children’ preferences nor their age but only some universal and intersubjective aspects of the music (i.e., tempo, rhythm, timbre, melody, scales and harmony), to favor its generalizability to the PICU environment.

All technical characteristics of the music followed the indications provided by previous literature [14,15,16]. In detail (further information available in Supplementary Materials), 432 Hz tuned music was specifically chosen for its physical and mathematical properties, which determine a more stable and harmonic sound [17,18,19]; and a frequency (tempo) of 70 beats per minute (bpm) was selected to favor the entrainment, which describes a process whereby two rhythmic processes interact with each other in such a way that they adjust towards and eventually “lock in” to a common phase or periodicity [20,21]. Considering the broad range of HR which we may encounter in a PICU, we chose 70 bpm as the frequency towards which individual HR could be pulled downwards, eliciting greater comfort.

Nurses managed the musical intervention, which consisted of 1 h of music played with a stable volume of approximately 50 decibels (limited to 60 decibels) by a stereo speaker system nearby the PICU beds, by allowing everyone (children, mothers and PICU staff) in the open-space room to listen to the music. The intervention was repeated for 1 h in the morning and 1 h in the afternoon, for the entire length of stay. Five tracks with styles, harmonies and timbres of musical instruments from both the western and oriental tradition were used as musical intervention. Two musical tracks (“Signs” and “Elephant”) had already been tested in previous clinical trials on adults [17,18,19] and one musical track (“Bliss”) was composed with the same criteria as those already tested, to be suitable for pediatric age. Finally, two songs (“Mother’s Mantra” and “Lullaby Melody”) were composed specifically for this project with the addition of a female voice in order to stimulate the cerebral neuroplasticity, activating a wide range of brain structures in children [22,23]. Before, during the musical intervention (at 30 min) and after 1 h, nurses recorded vital signs and filled the COMFORT Behavior Scale (CBS). No medical or nursing procedures were planned during the musical intervention. 

### 2.3. Data Collection

Baseline characteristics, including demographics and other information (i.e., religion), type of respiratory support (spontaneous breathing with oxygen therapy or mechanical ventilation), and vital signs such as HR, RR, blood pressure (BP) and oxygen saturation (SpO_2_) were collected by the trained nurse in charge. The main outcome measure was the sedation level evaluated using the CBS, which is a scale containing six behavioral dimensions: alertness, calmness or agitation, respiratory response or crying, physical movement, muscle tone and facial expression [24]. Each item is assessed based on a five-point Likert scale, from 1 (no distress) to 5 (severe distress). The total score ranged from 6 to 30 points, and each child was observed for 2 min. The cutoff points of the CBS were 10 and 23, where less than or equal to 10 represented oversedation and greater than or equal to 23 represented under sedation [25]. The CBS was introduced into clinical practice independently of the present study and, before starting data collection, a specific training was carried out on the PICU nursing staff. 

### 2.4. Statistical Analysis

Variables were summarized by median and interquartile range (IQR) or count and percentage (%). To evaluate the variation over time of the CBS, a mixed-effect regression model was fitted. The time-points selected were the three moments of assessment, namely before the intervention (Pre), after 30 and 60 min. Other time points were included as independent variables: days and moments (i.e., morning and afternoon) in which musical intervention was administered. The model was adjusted for the age of participants, which was represented by a three-knot restricted cubic spline function. Since each child was expected to have a unique reaction to pain, a random effect was included for participants; the model was fitted with an autocorrelation structure. The same approach was considered for estimating HR variation after and before musical intervention, as well as variation in other vital signs, considered as exploratory outcomes. The statistical analysis was performed using R Core Team (2019), version 3.6.1, with packages *lme4* added [26].

## 3. Results

Data were collected on 59 children admitted to PICU during the study period. Table 1 describes demographic characteristics at baseline. Since 23.7% (14/59) of children had more than six days of observation, analysis covered the first five days to further avoid unreliable estimates.

As detailed in Table 2, the majority of children were mechanically ventilated: 72.7% (24/33) were on pressure support ventilation and 27.3% (9/33) were on controlled ventilation. All children received analgesia (i.e., fentanyl or morphine) and sedation (i.e., midazolam or propofol) intravenously. Prior to initiation and during the music intervention, the dosage of administered drugs (rate of infusion or boluses) was not changed, and neuromuscular blocking agents were not administered.

Before the musical intervention, 46 children (78.0%) had an adequate sedation (CBS 11–22), 12 (20.3%) were over-sedated (CBS ≤ 10) and only 1 child (1.8%) was under-sedated (CBS ≥ 23). During the intervention all children had their mother next to the bed and no medical or nursing procedures were performed. 

**Figure 1:**
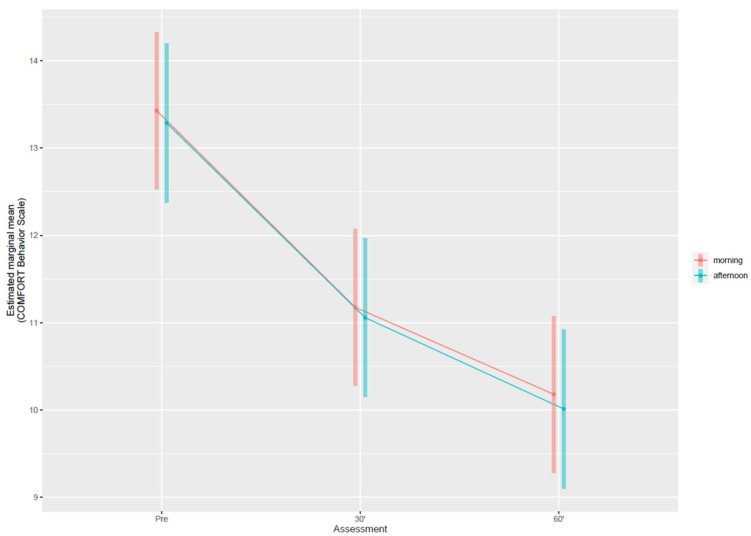
Mean variation of the COMFORT-Behavior Scale through assessment times (pre intervention, after 30 and 60 min), stratified by the moment of the day in which it occurred. Vertical bars denote 95% Confidence Interval.

Figure 1 shows the estimated CBS variation after the intervention. We found no evidence of association with the moment of the day (morning or afternoon) in which the musical intervention took place (0.1, 95% CI: −0.2 to 0.4 points). CBS was lowered by 2.2 (95% CI: 1.9 to 2.6) points after 30 min, and by 3.3 (95% CI: 2.9 to 3.6) points after 60 min from music initiation. Estimated drop between 30 and 60 min is 1 (95% CI: 0.7 to 1.4) point. Overall, we found evidence of association between CBS and days of PICU stay, with a variation of −1.3 (95% CI: −2.0 to −0.7) points after five days. Age of participants was not associated with the CBS variation (P = 0.568).

**Figure 2:**
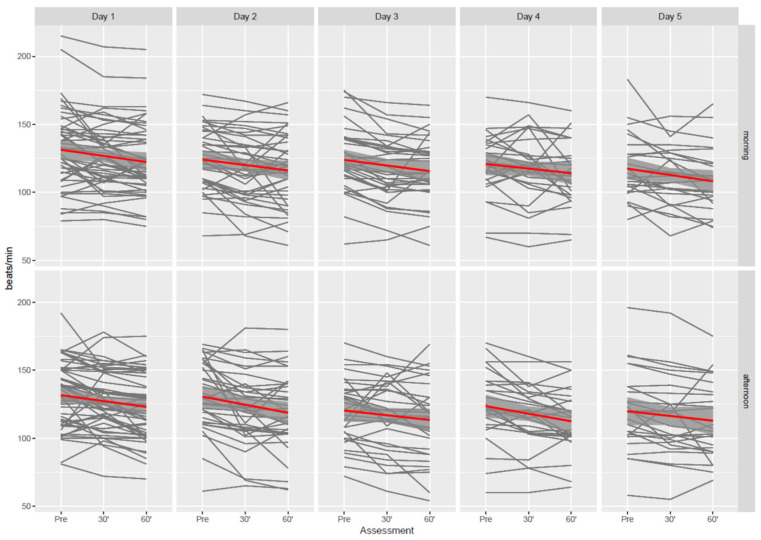
Time profiles of heart rate in the 59 participants from day 1 to day 5. Red solid line denotes regression line with 95% Confidence Intervals (grey bands).

HR time profiles for individual subjects stratified by days of observation are reported in Figure 2. Thirty minutes after music initiation, HR decreased by 6.3 (95% CI: 4.5 to 8.1) bpm, whereas at 60 min, HR decreased by 9.1 (95% CI: 7.2 to 10.9) bpm. No evidence of association was found between HR variation and morning/afternoon assessment (−1.0, 95% CI: −2.7 to 0.7 bpm). Contrary to the findings from the model for CBS, we found evidence of an association between age and HR (P < 0.001), suggesting a decrease in HR as age increased. Generally, HR decreased with the time children spent in PICU: after 5 days, HR reduced by 17.5 (95% CI: 11.9 to 23.1) bpm. Differences in the other vital signs (BP, RR and SpO_2_) are reported in Supplementary Materials. 

## 4. Discussion

This pilot study describes the implementation of a specific musical intervention in children admitted to PICU in a developing country. Our findings showed a meaningful reduction in CBS after 30 and 60 min from musical intervention, with no evidence of association with the moment of the day in which it took place. HR showed a decreasing trend after musical intervention as well, regardless the moment of the day and within limited range. Moreover, we reported differences in other vital signs after musical intervention; however, these were far from being clinically meaningful. 

Several studies have examined the positive responses to musical interventions in premature infants admitted to the neonatal ICU [27,28,29], but few involved pediatric patients. Previous experience showed that musical interventions in PICU were feasible and likely improved the comfort of children under mechanical ventilation [30], but no studies have been conducted so far in PICUs of developing countries. In fact, not all the interventions carried out in the ICUs of developed countries are feasible when adopted in clinical settings with less resources [31]. Musical intervention by listening to recorded music from a speaker is practical and much cheaper than listening to music live or through headphones, which are mostly used during clinical investigations [32]. The musical intervention adopted in this study is simple to perform at bedside, and nursing staff do not require specific training to implement it. Being highly reproducible, it can be incorporated into nursing care even when resources are scarce [32] and there is little chance to consider patients’ preference. 

Our findings showed a meaningful variation (i.e., CBS ≥ 2) of level of sedation assessed with CBS: although this is a pilot observational study, the results support the use of music as an inexpensive and non-pharmacologic intervention to elicit a change in CBS and HR, that is worth further exploration. Moreover, our study shares similar results with other studies conducted in different countries [30,33,34]. A recent study conducted in China demonstrated that CBS of 50 children admitted to PICU was lower in the group exposed to musical intervention (CBS variation was by −2.1 points after 60 min), indicating more comfort compared to the control group [30]. Another study conducted in 84 children from Brazil looked at the use of classical music in PICU following heart surgery, demonstrating that musical intervention had a significant beneficial effect during the postoperative period, evaluated using both a facial pain scale and HR [34]. The physiological aspect of HR reduction is in agreement with other investigations that have also registered a variation after musical intervention [35]. Music induces effects based on autonomic responses: for this reason, it can slow down the RR and modulate autonomous cardiovascular regulation [36,37], eventually reducing HR. These results are also in line with the assumption that music with a slow steady rhythm provides stress reduction by altering inherent body rhythms, such as the HR [38]. Overall, the benefits of any musical intervention could have a positive impact by mitigating stress symptoms; however, no clear clinically important differences are available so far in the considered literature to determine a positive response after musical intervention. 

Regarding other vital signs, which showed a statistically significant variation, few considerations about lack of clinical relevance can be made. Of the children, 44.1% were breathing spontaneously, whereas the others had some type of respiratory support with oxygen supplementation. Data collection was not as accurate as to describe ventilatory modes or to quantify oxygen prescription; however, we can think that respiratory support was well managed, thus showing on average a small variation in both RR and SpO_2_. By consequence, during musical interventions in PICU, relying on SpO_2_ or RR might be a poor choice due to the presence of confounding. It is intuitive thinking that stress affects BP as well; however, inotropic support and sedation affect BP too, which were not recorded in the present study. Validity of the 3–7 mmHg drop seen in our study should be interpreted with caution; however, it is important to note that such variation is greater than one reported by the pilot randomized controlled trial of Liu et al. (i.e., −2.4 and −3.8 mmHg for systolic and diastolic BP, respectively) [30].

The choice of music pieces is the cornerstone of any therapeutic listening [32]. The tracks must be specifically composed taking into consideration the setting (the hospital wards are different from each other), the age (children, as opposed to newborns or adults) and the cultural background of those who listen (choice of instruments, styles and genres) [39]. Particularly, despite neurobiological traits for musicality being shared among all humans, cultural variability and uniqueness of each musical event might have a role towards an effective musical intervention played locally. Independently from children’s preferences, the musical tracks used in the present study considered universal and intersubjective aspects of the music to favor its generalizability.

Unlike previous studies, this research was carried out in Iraqi Kurdistan, a socio-cultural context where the children are predominantly of Arabic culture (Muslim and Yazidi). The essence of traditional Islamic music is contained in the notes themselves, in scales that exploit the vast expressive possibilities of microtonality, using notes not used in Western music [40]. Indeed, microtones (i.e., *shruti*) derived by bansuri flute and voice were used in the music tracks. The melodic structures of traditional Islamic music allow for the communication of an infinite variety of human emotions and spiritual attitudes. However, even more important than the notes themselves are the ways in which their understanding is conveyed [40]. Nevertheless, the present findings yielded similar and positive results in terms of CBS and HR, as collected from other experiences from developed countries.

The majority of musical interventions in children were mainly conducted out of PICUs, and all described a significant reduction in pain, anxiety and distress before, during or after a minor or major surgery [41] or in the emergency department, particularly for children undergoing intravenous placement [42]. Even in a large meta-analysis [43], few studies (only five RCTs) included children, and all the interventions were performed outside PICUs. Evidence suggests that musical interventions should be considered for clinical use as non-pharmacological interventions in different wards; furthermore, listening to music through speakers could also benefit the parents and the ICU staff [42]. Therefore, considered the paucity of studies, further investigations in different contexts and cultures are necessary to deepen the use and effectiveness of the musical intervention in the routine care of PICU.

### Strengths and Limitations

Several study limitations need to be addressed. We did not use a control group so changes in outcomes should not be interpreted under causality. Blinding of nurses assessing study outcomes was not possible, thus outcome assessment could be influenced by nurses. Formally, we did not explore feasibility, conceived as how nurses experienced the delivery of the musical intervention. However, staff considered such initiative generally positive, especially because it did not increase workload nor required additional training. Moreover, we could not record the exact noise volume in the PICU nor the distance between the stereo speakers and children’ ears. Contrary to the current suggestions, we did not use headphones during the musical intervention [32].

Despite these limitations, we have provided initial impression of CBS and HR variation following a non-pharmacologic treatment. Narrow confidence intervals and medium-to-large effect sizes in CBS variation at 30 (approximate Cohen’s d = 0.78) and at 60 min (d = 1.1) are promising to look further into musical intervention in PICU as conceived in the present study, which used specific characteristics that are unique in the literature. Furthermore, setting the smallest effect size to a CBS variation of 2 within our data, we obtained a statistical power of 100 (95% CI: 99.2 to 100.0) %, using 500 Monte Carlo simulation and SIMR package [44], thus making our findings reliable.

## 5. Conclusions

This pilot study supports the application of a tailored music intervention that plausibly changes the level of sedation in children admitted to PICU. As a non-pharmacologic therapy, music can be individualized according to cultural variability, argued to evoke pleasant nervous-mediated responses. Most importantly, music remains a low-cost intervention, particularly suitable in developing countries. 

Further studies are needed to provide conclusive evidence about the efficacy of musical therapy in critically ill children in PICU, possibly adjusting for concomitant medical therapies. Definition of clinically important differences in the behavioral and physiologic parameters considered so far in literature remains a crucial point for future projects on musical interventions in PICU.

## Figures and Tables

**Table 1:** Demographic and clinical characteristics of the patients.

Characteristics	N = 59
Age (years)	2.0 (0.7–8.0)
Sex	
Male	35 (59.0%)
Female	24 (41.0%)
Religion	
Muslim	49 (83.0%)
Yazidism	10 (17.0%)
Admission Diagnosis	
Gastrointestinal	18 (30.5%)
Neurologic	15 (25.4%)
Cardiovascular	7 (11.9%)
Trauma	7 (11.9%)
Nephrological	4 (6.8%)
Oncologic	1 (1.7%)
Respiratory	1 (1.7%)
Other diagnosis	6 (10.2%)

**Table 2:** Respiratory support and vital signs at baseline.

Respiratory Support	N = 59
Spontaneous breathing	26 (44.1%)
High flow nasal cannula	18 (30.5%)
Oxygen mask	8 (13.6%)
Mechanical ventilation	33 (55.9%)
Endotracheal tube	30 (50.8%)
Tracheostomy tube	3 (5.1%)
**Vital Signs**	
Heart rate (beats per minute)	132.0 (114.0–148.0)
Systolic blood pressure (mmHg)	114.0 (103.0–124.0)
Diastolic blood pressure (mmHg)	64.0 (57.0–76.0)
Respiratory rate (breaths per minute)	32.0 (29.0–41.0)
Oxygen saturation (%)	100.0 (98.0–100.0)

## Data Availability

The data presented in this study are available on request from the corresponding author.

## Supplementary Materials

The following supporting information can be downloaded at: https://www.mdpi.com/article/10.3390/children9040455/s1, Figure S1: Time profiles of Respiratory Rate in the 59 participants from day 1 to day 5; Figure S2: Time profiles of Oxygen Saturation in the 59 participants from day 1 to day 5; Figure S3: Time profiles of Systolic Blood Pressure in the 59 participants from day 1 to day 5; Figure S4: Time profiles of Diastolic Blood Pressure in the 59 participants from day 1 to day 5; Table S1: Estimates of mixed-effect models; Table S2: Musical intervention details.

## References

[1] Ciğerci Y., Kısacık Ö.G., Özyürek P., Çevik C. (2019). Nursing music intervention: A systematic mapping study. Complement. Ther. Clin. Pract..

[2] Drahota A., Ward D., Mackenzie H., Stores R., Higgins B., Gal D., Dean T.P. (2012). Sensory environment on health-related outcomes of hospital patients. Cochrane Database Syst. Rev..

[3] Mrázová M., Celec P. (2010). A Systematic Review of Randomized Controlled Trials Using Music Therapy for Children. J. Altern. Complement. Med..

[4] Hu R.F., Jiang X.Y., Chen J., Zeng Z., Chen X.Y., Li Y., Huining X., Evans D.J.W. (2015). Non-pharmacological interventions for sleep promotion in the intensive care unit. Cochrane Database Syst. Rev..

[5] Messika J., Martin Y., Maquigneau N., Puechberty C., Henry-Lagarrigue M., Stoclin A., Panneckouke N., Villard S., Dechanet A., Lafourcade A. (2019). A musical intervention for respiratory comfort during noninvasive ventilation in the ICU. Eur. Respir. J..

[6] Kaur H., Rohlik G., Nemergut M., Tripathi S. (2016). Comparison of staff and family perceptions of causes of noise pollution in the Pediatric Intensive Care Unit and suggested intervention strategies. Noise Health.

[7] Weatherhead J.R., Niedner M., Dahmer M.K., Malas N., Owens T., Kawai Y. (2021). Patterns of Delirium in a Pediatric Intensive Care Unit and Associations with Noise Pollution. J. Intensive Care Med..

[8] Smith J.R., Pineda R.G. (2016). Determining Appropriate Sensory Exposures in the NICU: Too Much, Too Little, or Just Right?. Neonatal Netw..

[9] Ko M.S.M., Poh P.-F., Heng K.Y.C., Sultana R., Murphy B., Ng R.W.L., Lee J.H. (2022). Assessment of Long-term Psychological Outcomes After Pediatric Intensive Care Unit Admission: A Systematic Review and Meta-analysis. JAMA Pediatr..

[10] Foroushani S.M., Herman C.A., Wiseman C.A., Anthony C.M., Drury S.S., Howell M.P. (2020). Evaluating physiologic outcomes of music interventions in the neonatal intensive care unit: A systematic review. J. Perinatol..

[11] Tripathi S., Kaur H., Kashyap R., Dong Y., Gajic O., Murthy S. (2015). A survey on the resources and practices in pediatric critical care of resource-rich and resource-limited countries. J. Intensive Care.

[12] Robb S.L., Carpenter J.S., Burns D.S. (2011). Reporting guidelines for music-based interventions. J. Health Psychol..

[13] United Nations High Commissioner for Refugees (2021). Syria Emergency. https://www.unhcr.org.

[14] Bernardi L., Porta C., Sleight P. (2006). Cardiovascular, cerebrovascular, and respiratory changes induced by different types of music in musicians and non-musicians: The importance of silence. Heart.

[15] Bernardi L., Porta C., Casucci G., Balsamo R., Bernardi N.F., Fogari R., Sleight P. (2009). Dynamic interactions between musical, cardiovascular, and cerebral rhythms in humans. Circulation.

[16] Khalfa S., Roy M., Rainville P., Dalla Bella S., Peretz I. (2008). Role of tempo entrainment in psychophysiological differentiation of happy and sad music?. Int. J. Psychophysiol..

[17] Modesti P.A., Ferrari A., Bazzini C., Costanzo G., Simonetti I., Taddei S., Biggeri A., Parati G., Gensini G.F., Sirigatti S. (2010). Psychological predictors of the antihypertensive effects of music-guided slow breathing. J. Hypertens..

[18] Modesti P.A., Ferrari A., Bazzini C., Boddi M. (2015). Time sequence of autonomic changes induced by daily slow-breathing sessions. Clin. Auton. Res..

[19] Di Nasso L., Nizzardo A., Pace R., Pierleoni F., Pagavino G., Giuliani V. (2016). Influences of 432 Hz Music on the Perception of Anxiety during Endodontic Treatment: A Randomized Controlled Clinical Trial. J. Endod..

[20] Bernardi N.F., Codrons E., di Leo R., Vandoni M., Cavallaro F., Vita G., Bernardi L. (2017). Increase in Synchronization of Autonomic Rhythms between Individuals When Listening to Music. Front. Physiol..

[21] Clayton M., Sager R., Will U. (2005). In time with the music: The concept of entrainment and its significance for ethnomusicology. Eur. Meet. Ethnomusicol..

[22] Filippa M., Lordier L., De Almeida J.S., Monaci M.G., Adam-Darque A., Grandjean D., Kuhn P., Hüppi P.S. (2020). Early vocal contact and music in the NICU: New insights into preventive interventions. Pediatr. Res..

[23] An K., Hasegawa C., Hirosawa T., Tanaka S., Saito D.N., Kumazaki H., Yaoi K., Kikuchi M., Yoshimura Y. (2020). Brain responses to human-voice processing predict child development and intelligence. Hum. Brain Mapp..

[24] Ista E., Van Dijk M., Tibboel D., De Hoog M. (2005). Assessment of sedation levels in pediatric intensive care patients can be improved by using the COMFORT “behavior” scale. Pediatr. Crit. Care Med..

[25] Vet N.J., Kleiber N., Ista E., de Hoog M., de Wildt S.N. (2016). Sedation in Critically Ill Children with Respiratory Failure. Front. Pediatr..

[26] R Core Team (2019). R: A Language and Environment for Statistical Computing. https://www.r-project.org.

[27] Alipour Z., Eskandari N., Ahmari Tehran H., Eshagh Hossaini S.K., Sangi S. (2013). Effects of music on physiological and behavioral responses of premature infants: A randomized controlled trial. Complement. Ther. Clin. Pract..

[28] Taheri L., Jahromi M.K., Abbasi M., Hojat M. (2017). Effect of recorded male lullaby on physiologic response of neonates in NICU. Appl. Nurs. Res..

[29] Schwilling D., Vogeser M., Kirchhoff F., Schwaiblmair F., Boulesteix A.-L., Schulze A., Flemmer A.W. (2015). Live music reduces stress levels in very low-birthweight infants. Acta Paediatr..

[30] Liu M.H., Zhu L.H., Peng J.X., Zhang X.P., Xiao Z.H., Liu Q.J., Qiu J., Latour J.M. (2020). Effect of Personalized Music Intervention in Mechanically Ventilated Children in the PICU: A Pilot Study. Pediatr. Crit. Care Med..

[31] Turner H.C., Hao N.V., Yacoub S., Hoang V.M.T., Clifton D.A., Thwaites G.E., Dondorp A.M., Thwaites C.L., Chau N.V.V. (2019). Achieving affordable critical care in low-income and middle-income countries. BMJ Glob. Health.

[32] Robb S.L., Hanson-Abromeit D., May L., Hernandez-Ruiz E., Allison M., Beloat A., Daugherty S., Kurtz R., Ott A., Oyedele O.O. (2018). Reporting quality of music intervention research in healthcare: A systematic review. Complement. Ther. Med..

[33] Kennelly J., Queensland H., Edwards J. (1997). Providing music therapy to the unconscious child in the paediatric intensive care unit. Aust. J. Music Ther..

[34] Hatem T.P., Lira P.I.C., Mattos S.S. (2006). The therapeutic effects of music in children following cardiac surgery. J. Pediatr..

[35] Garcia Guerra G., Joffe A.R., Sheppard C., Hewson K., Dinu I.A., Hajihosseini M., deCaen A., Jou H., Hartling L., Vohra S. (2021). Music Use for Sedation in Critically ill Children (MUSiCC trial): A pilot randomized controlled trial. J. Intensive Care.

[36] Liu Y., Petrini M.A. (2015). Effects of music therapy on pain, anxiety, and vital signs in patients after thoracic surgery. Complement. Ther. Med..

[37] Wu P.-Y., Huang M.-L., Lee W.-P., Wang C., Shih W.-M. (2017). Effects of music listening on anxiety and physiological responses in patients undergoing awake craniotomy. Complement. Ther. Med..

[38] Thaut M.H., Hoemberg V. (2014). Handbook of Neurologic Music Therapy.

[39] Fitzsimons B. (2016). Approaching music therapy in a different country: A literature review on cultural considerations when practising in a developing country. Br. J. Music Ther..

[40] What Is Islamic Music?|The Muslim 500. https://themuslim500.com/guest-contributions-2016/what-is-islamic-music/.

[41] Van Der Heijden M.J.E., Araghi S.O., Van Dijk M., Jeekel J., Hunink M.G.M. (2015). The Effects of Perioperative Music Interventions in Pediatric Surgery: A Systematic Review and Meta-Analysis of Randomized Controlled Trials. PLoS ONE.

[42] Hartling L., Newton A.S., Liang Y., Jou H., Hewson K., Klassen T.P., Curtis S. (2013). Music to Reduce Pain and Distress in the Pediatric Emergency Department: A Randomized Clinical Trial. JAMA Pediatr..

[43] Lee J.H. (2016). The Effects of Music on Pain: A Meta-Analysis. J. Music Ther..

[44] Green P., Macleod C.J. (2015). SIMR: An R package for power analysis of generalized linear mixed models by simulation. Methods Ecol. Evol..

