# Supplementary material for: Use of Musical Intervention in the Pediatric Intensive Care Unit of a Developing Country: A Pilot Pre–Post Study
Source: Children (Basel). 2022 Mar 24;9(4):455. doi: 10.3390/children9040455 (PMC9032317; doi:10.3390/children9040455)
Supplement: Supplementary file 1 [file children-09-00455-s001.zip › children-1559541-supplementary.pdf]

Supplementary Materials

Use of Musical Intervention in the Pediatric Intensive Care Unit of a Developing Country: A Pilot Pre–Post Study

Federica Buzzi, Nizar Bakir Yahya, Simone Gambazza, Filippo Binda, Alessandro Galazzi, Antonella Ferrari, Stefano Crespan, Hevan Adel Al-Atroushy, Barbara Maria Cantoni and Dario Laquintana and Collaborative Group †

† Bayar Saleem Haji, Shaima Shimo Jaafar, Sami Gabriel Saeb, Loreen Ahmed Haji, Muhamed Malla Awni, Ali Mohammed Saeed, Bijeen Fareq Jalal, Bushra Mosa Abdulrahman, Nalin Saed Sabah, Sholker Khalid Mohammad, Ikhlass Ramadhan Taha.

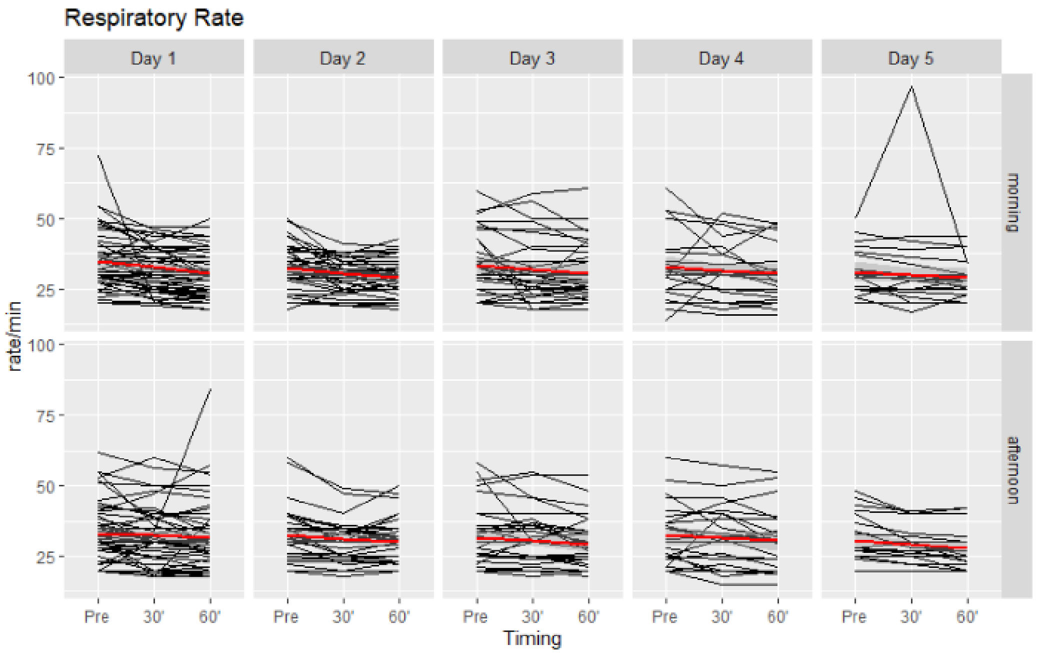

Figure S1. Time profiles of Respiratory Rate in the 59 participants from day 1 to day 5. Red solid line denotes regression line with 95% Confidence Intervals (grey bands). Estimated variation at 30′ is -2.0 (95%CI: -2.7 to -1.2) rate/min, whereas at 60′ is -2.6 (95%CI: -3.3 to -1.8) rate/min.

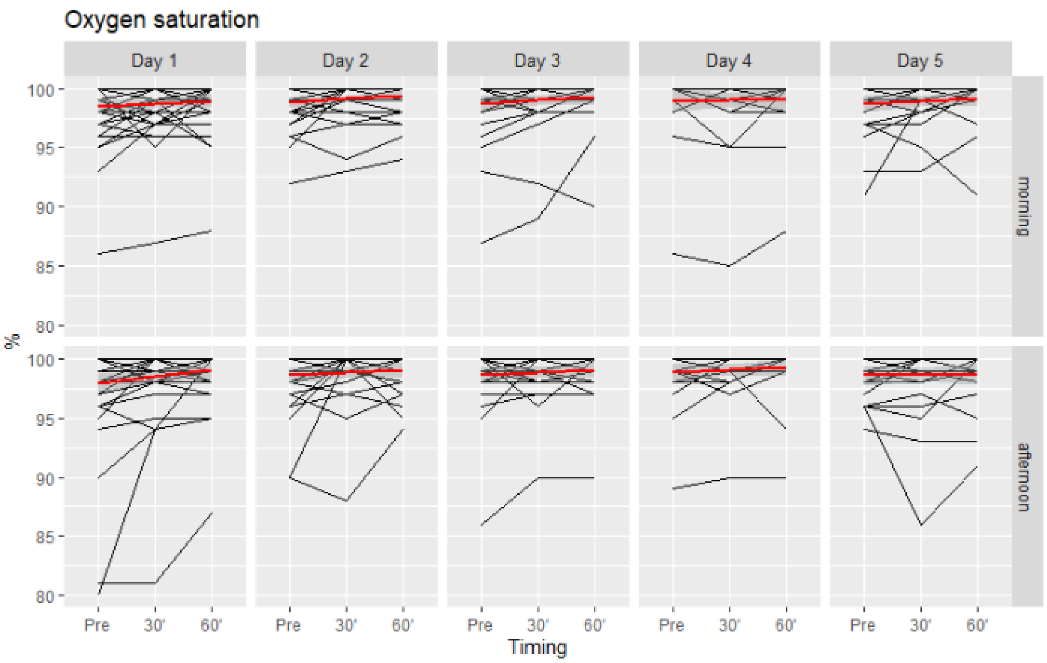

Figure S2. Time profiles of Oxygen Saturation in the 59 participants from day 1 to day 5. Red solid line denotes regression line with 95% Confidence Intervals (grey bands). Estimated variation at 30′ is 0.3 (95%CI: 0.1 to 0.5) %, whereas at 60′ is 0.5 (95%CI: 0.3 to 0.7) %.

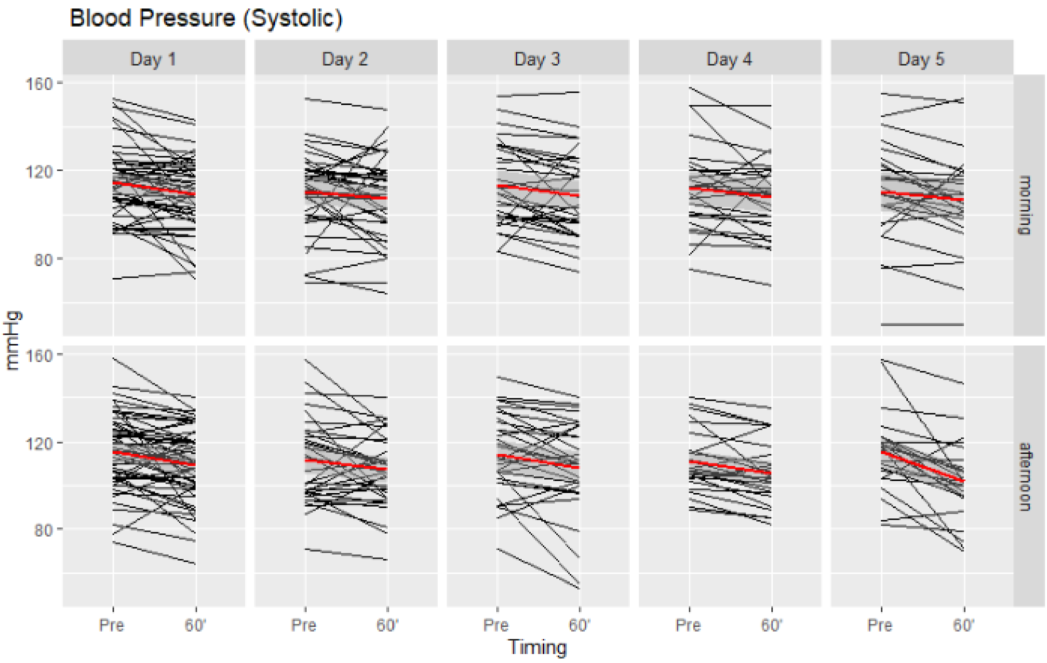

Figure S3. Time profiles of Systolic Blood Pressure in the 59 participants from day 1 to day 5. Red solid line denotes regression line with 95% Confidence Intervals (grey bands). Estimated variation at 60′ is -5.5 (95%CI: -7.0 to -4.0) mmHg.

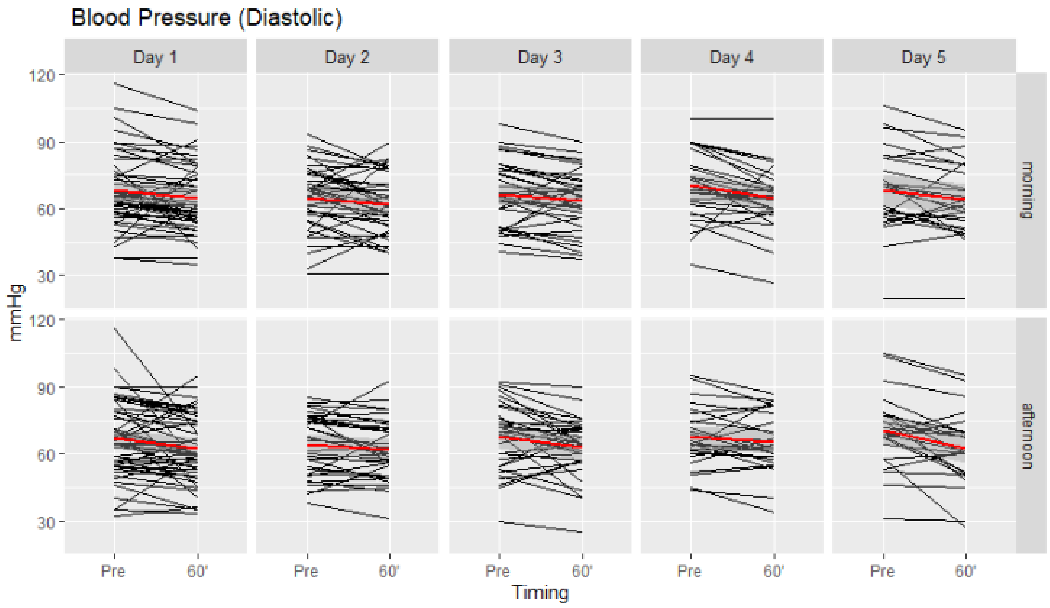

Figure S4. Time profiles of Diastolic Blood Pressure in the 59 participants from day 1 to day 5. Red solid line denotes regression line with 95% Confidence Intervals (grey bands). Estimated variation at 60′ is -4.0 (95%CI: -5.2 to -2.7) mmHg.

Table S1. Estimates for the mixed-effect models

	COMFORT-Behavior Scale			Heart Rate			Respiratory Rate			Oxygen Saturation			Systolic Blood Pressure			Diastolic Blood Pressure		
	**Estimate**	**SE**	**P-value**	**Estimate**	**SE**	**P-value**	**Estimate**	**SE**	**P-value**	**Estimate**	**SE**	**P-value**	**Estimate**	**SE**	**P-value**	**Estimate**	**SE**	**P-value**
**Intercept**	13.3	0.59	<0.001	142.37	5.64	<0.001	42.75	1.77	<0.001	98.31	0.53	<0.001	105.14	3.94	<0.001	58.63	3.33	<0.001
**Moments of assessment**			<0.001			<0.001			<0.001			<0.001			<0.001			<0.001
Before	-			-			-			-			-			-		
30' assessment	-2.24	0.15		-6.29	0.78		-1.99	0.39		0.30	0.10		-			-		
60' assessment	-3.27	0.15		-9.06	0.79		-2.59	0.39		0.5	0.10		-5.49	0.77		-3.96	0.65	
**Day timing**			0.339			0.243			0.154			0.082			0.345			0.458
Morning	-			-			-			-			-			-		
Afternoon	-0.14	0.15		1.02	0.87		-0.56	0.39		0.18	0.10		0.85	0.90		0.55	0.75	
**Days of assessment**			<0.001			<0.001			<0.001			0.106			0.778			0.670
Day 1	-			-			-			-			-			-		
Day 2	-0.11	0.27		-8.57	1.98		-3.77	0.74		0.48	0.19		-0.56	1.83		0.72	1.47	
Day 3	-0.54	0.29		-14.02	2.4		-3.49	0.83		0.31	0.22		1.37	2.09		1.83	1.66	
Day 4	-0.39	0.32		-15.62	2.66		-3.55	0.89		0.47	0.23		0.47	2.28		4.38	1.80	
Day 5	-1.32	0.33		-17.51	2.87		-4.58	0.93		0.19	0.24		-1.01	2.39		2.91	1.88	
**Age**			0.568			<0.001			<0.001			0.949			<0.001			<0.001
Spline 1	0.72	1.30		-25.57	11.15		-6.80	3.80		0.53	1.11		21.45	8.41		20.75	7.11	
Spline 2	0.33	1.34		-28.90	12.89		-21.77	4.02		0.40	1.21		23.80	8.85		21.20	7.53	
Spline 3	-1.08	0.91		-33.17	8.04		-11.37	2.67		-0.06	0.78		6.82	5.96		5.44	5.02	

Table S2. Musical intervention details

Name	Time	Instruments	Voice
Signs	15′ 07″	Piano, strings, bansuri flute, harp, tampura, percussions	no
Elephant	12′ 10″	Piano, strings, bansuri flute, tampura, percussions	no
Bliss	13′ 20″	Piano, strings, bansuri flute, sitar, tampura	yes
Mother's mantra	10′ 30″	Piano, strings, bansuri flute, duduk, percussions	yes
Lullaby Melody	09′ 10″	Piano, keyboards	yes

Copyright© Shantam Stefano Crespan and Deva Sound 2008-2022. All the demo tracks are available for listening on the website: www.curadelsuono432.com
